# Distant embolisation in infective endocarditis: characteristics and outcomes

**DOI:** 10.1007/s12471-023-01771-6

**Published:** 2023-03-30

**Authors:** Mariëlle G. J. Duffels, Tjeerd Germans, Annet Bos-Schaap, Olivier Drexhage, Jiri F. P. Wagenaar, Friso M. van der Zant, Martine Hoogewerf, Remco J. J. Knol, Victor A. W. M. Umans

**Affiliations:** 1 Department of Cardiology, Northwest Clinics, Alkmaar, The Netherlands; 2 Department of Infectious Diseases, Northwest Clinics, Alkmaar, The Netherlands; 3 Department of Nuclear Medicine, Northwest Clinics, Alkmaar, The Netherlands; 4 Department of Medical Microbiology, Northwest Clinics, Alkmaar, The Netherlands

**Keywords:** Infective endocarditis, Embolisation, Imaging, Clinical cardiology

## Abstract

**Background:**

Infective endocarditis is a severe and potentially lethal cardiac disease. Recognition of the clinical features of endocarditis, such as distant embolisation, and adequate treatment should be initiated promptly given the grim perspective of upcoming virulent pathogens.

**Methods:**

We report on our registry-based experience with outcomes of consecutive patients with infective endocarditis with distant embolisation. We aimed to describe the patient characteristics of infective endocarditis complicated by distant organ embolisation and the safety aspects of continuing endocarditis treatment at home in these patients.

**Results:**

From November 2018 through April 2022, 157 consecutive patients were diagnosed with infective endocarditis. Of them, 38 patients (24%) experienced distant embolisation, either in the cerebrum (*n* = 18), a visceral organ (*n* = 5), the lungs (*n* = 7) or the myocardium (*n* = 8). Pathogens identified in blood cultures were predominantly streptococcal variants (43%), with only one culture-negative endocarditis case. Of the 18 patients with cerebral embolisation, 12 had neurological complaints and most often discrete abnormal findings on neurological examination. Six of the 8 cardiac embolism patients experienced chest pain before admission. Visceral organ and pulmonary embolism occurred silently. Of the 38 patients with distant embolisation, 17 could be discharged earlier by providing antibiotic treatment at home without complications.

**Conclusion:**

This registry-based single-centre experience showed an incidence of distant embolisation in daily care of 24%. Cerebral and coronary embolisation provoked symptoms, while visceral emboli remained silent. Pulmonary emboli may present with inflammatory signs. Distant embolisation was not in itself a contra-indication for outpatient endocarditis@home treatment.

## What’s new?

- This registry-based, single-centre experience showed an incidence of distant embolisation in a non-referral hospital of 24%.
- With regard to the occurrence of distant embolisation, there are three important aspects: it may establish the diagnosis of endocarditis, impact clinical decision-making and affect the clinical and inflammatory course of the disease.
- Patients with distant organ embolic events may be seen at the internal medicine, orthopaedics or neurology department with localised symptoms and a common denominator of positive blood cultures while being febrile or septic.
- Outpatient intravenous antibiotic treatment is also feasible for recovering patients with distant organ embolisation and may warrant adjustment of the guidelines.

## Introduction

Infective endocarditis is a common cardiac disease with a potential fatal clinical course [1, 2]. The natural course of endocarditis has changed, with different pathogens and patient characteristics [1, 3–5]. The incidence has been reported to increase over time, with a rise in more virulent microorganisms in earlier studies [3–5]. The prevalence of infective endocarditis is estimated to be 3–10 per 100,000 inhabitants.

Although several patient groups at risk have been identified, i.e. patients with mechanical cardiac valves or cardiovascular implantable devices, the majority of endocarditis patients are non-high-risk patients [3, 6]. However, recognition and treatment of endocarditis have become more sophisticated, and adequate treatment should be initiated promptly given the grim perspective of upcoming virulent pathogens. This is even more apparent in patients with distant embolic events either at presentation or during the course of their treatment because this requires more sophisticated recognition and treatment [5–9]. Such patients may be seen, for example, at the internal medicine, orthopaedics or neurology department with localised symptoms and a common denominator of positive blood cultures while being febrile [10–13]. A multidisciplinary approach should entail an imminent multi-imaging workup and the start of effective and culture-driven antibiotic therapy.

We aimed to describe the patient characteristics of infective endocarditis complicated by distant organ embolisation in a non-surgical hospital. Additionally, we provide information on the safety aspects of continuing endocarditis treatment at home in these patients.

## Methods

### Patient population

From November 2018 through April 2022, all patients with suspected endocarditis were presented at the weekly Endocarditis Team meeting at Northwest Clinics in Alkmaar, the Netherlands. Confirmation or rejection of the diagnosis of infective endocarditis was based on clinical presentation, blood culture sampling and multimodality imaging. All consecutive patients with a confirmed diagnosis were prospectively followed and constituted the patient cohort.

Infective endocarditis was defined according to the 2015 European Society for Cardiology (ESC) modified diagnostic criteria [1]. Multimodality imaging included transthoracic echocardiography and/or transoesophageal echocardiography. When indicated, ^18^F‑fluorodeoxyglucose (FDG) positron emission tomography (PET)/computed tomography (CT) (*n* = 91) or cardiac magnetic resonance imaging (MRI) (*n* = 8) was performed.

Embolic events were defined as (sub)clinical wedge-shaped lesions shown on any imaging modality. These were subdivided into cardiac, pulmonal, visceral organ and cerebral embolisms.

### Endocarditis team

Endocarditis presentations may result in admissions and treatments by medical specialists from diverse disciplines, which often delays the diagnosis and the initiation of optimal therapy. We therefore formed the Alkmaar Endocarditis Team in accordance with the ESC Guidelines [1]. In Endocarditis Team meetings, we discuss the following patient groups: (a) patients in whom the diagnosis is not yet confirmed and (b) patients with a stable clinical course while on appropriate antibiotic treatment. The team, with in-person expertise of (imaging) cardiologists, infectious disease specialists, nurse practitioners, microbiologists and nuclear radiologists, meets weekly.

Cardio-surgical expertise is organised in a stepwise fashion. Firstly, the surgeon comes to our clinic weekly and may visit endocarditis patients on demand to ascertain operability. Secondly, patients with a complicated disease course are presented in the tertiary Endocarditis Team meeting via video call. Finally, the surgeons are always on call in case of emergency needs. Meeting notes and treatment plans are registered in the patient’s electronic medical record. The nurse practitioners provide clinical follow-up and are responsible for data registration.

### Data retrieval

Patient data, such as demographics, medical history, diagnostics, microbiological culture results, antibiotic treatment and complications during admission and follow-up, were collected. All data were retrieved electronically from the electronic medical records. Outcome parameters were the occurrence of organ embolisation and relapse at 30 days.

### Statistical analysis

Continuous variables are presented as mean ± standard deviation. Categoric variables are presented as absolute numbers, and percentages where applicable. Continuous variables were compared by means of the Student’s paired *t*-test where applicable.

## Results

### Patient characteristics

From November 2018 through April 2022, 157 consecutive patients were diagnosed with infective endocarditis. They were predominantly male (65%), and the mean age was 72 ± 12 years. C‑reactive protein (CRP) and leucocyte levels at admission were 127 ± 95 mg/l and 12.4 ± 7.0 × 10^9^/l, respectively. Cultured pathogens were predominantly streptococcal variants (44%), while 4% of the cultures were negative. The predominantly affected valve was the aortic valve. Of the 157 endocarditis patients, 65% had prosthetic valve endocarditis. Pacemaker or implantable cardioverter-defibrillator (ICD) lead or device infections were demonstrated in 30 patients (19%). Mean length of hospital stay was 46 ± 23 days.

### Patients with distant organ emboli

**Fig. 1:**
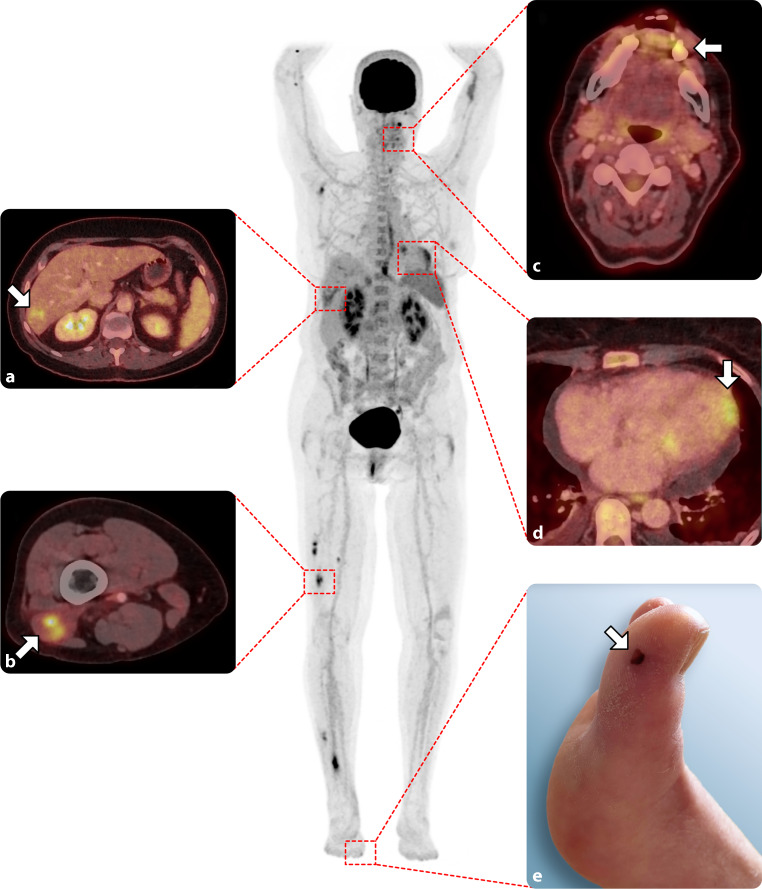
Total body maximum intensity projection with slices of fused ^18^F‑fluorodeoxyglucose positron emission tomography/computed tomography images showing embolisation to **a** right liver lobe, **b** biceps femoris muscle, **c** left mandibula (with possible primary (dental) focus) and **d** left ventricular apex. **e** Discrete focal FDG uptake is present in Jane Way lesion on medial side of right big toe

**Fig. 2:**
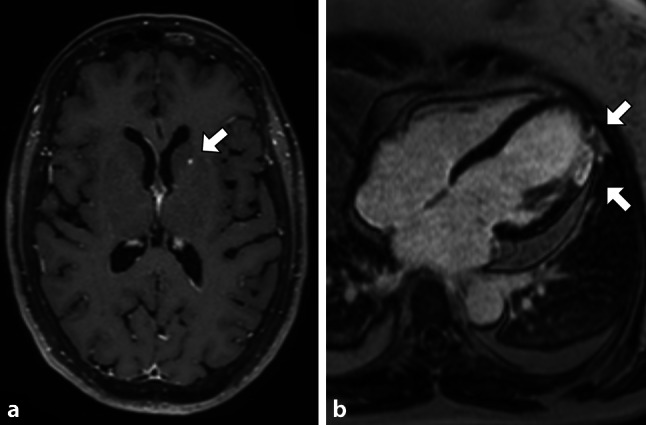
Magnetic resonance imaging. **a** Section of cerebrum showing embolism in left internal capsule (*see arrow*). **b** Section of heart with transmural apical late enhancement with microvascular obstruction (*see arrows*) corresponding with recent transmural infarction

Of the 157 patients included, 38 (24%) experienced distant organ embolisation in the cerebrum, a visceral organ (kidney, spleen, liver), the lungs or the myocardium (Figs. 1 and 2). Four patients presented with multiple organ embolisation, and none had soft tissue abscesses. Patients with embolism were predominantly male (58%) and had a mean age of 73 ± 12 years (Tab. 1). CRP and leucocyte levels at admission were 135 ± 97 mg/l. and 12.7 ± 5.4 × 10^9^/l, respectively. The pathogens identified in blood cultures were predominantly streptococcal variants (43%), and there was one culture-negative endocarditis case.

**Table 1:** Demographic characteristics of 157 endocarditis patients

Characteristic	Embolism (*n* = 38)	No embolism (*n* = 119)	*P-*value
Age, years	73 ± 12	75 ± 12	0.684
Female	16 (42)	38 (32)	0.270
**Pathogens**			0.263
*Streptococcus*	17 (44)	51 (42)	
*Enterococcus*	8 (21)	20 (17)	
*Staphylococcus*	11 (29)	32 (27)	
Negative culture	1 (3)	5 (4)	
Others	1 (3)	11 (10)	
**Laboratory results**			
C‑reactive protein, mg/l	135	124	0.483
Haemoglobin, mmol/l	7.4	7.0	0.226
Leucocytes, × 10−9/l	12.7	12.3	0.709
Creatinine, μmol/l	128	124	0.949
**Cardiac involvement**			0.468
Native valve	17 (44)	54 (45)	
Prosthetic valve	12 (32)	28 (24)	
Aortic prothesis	0	4 (3)	
Pacemaker/ICD	8 (21)	22 (18)	
Other/unknown location	1 (3)	12 (10)	

Data are mean ± standard deviation, *n* (%) or mean

*ICD* implantable cardioverter-defibrillator

The predominantly affected valve was the aortic valve. Of the 38 endocarditis patients with distant organ metabolism, 12 had prosthetic valve endocarditis and in 1 case, the infected valve could not be characterised definitively. Device lead infections were demonstrated in 7 of the 38 patients. Mean duration of in-hospital stay was 54 ± 36 days.

#### Cerebrovascular embolism

In 18 patients, evidence of a cerebrovascular accident was found. Of these patients, 12 had neurological complaints and most often discrete abnormal findings on neurological examination, and 7 had signs of cerebral embolism on neurologic examination or on cerebral imaging within 24 h of presentation. The aortic valve was affected in 7 patients, of whom 1 suffered from visceral organ and definite coronary embolism and 5 were diagnosed with spondylodiscitis. Microbiological cultures revealed the presence of *Staphylococcus aureus* (*n* = 4), coagulase-negative* Staphylococcus* (*n* = 1), streptococcal variants (*n* = 7) and *Enterococcal species* (*n* = 6). One patient underwent urgent valve replacement. In-hospital mortality occurred in 3 patients secondary to the distant embolisation.

Seven patients, including the patient who underwent urgent valve replacement, could be enrolled in successful and uncomplicated outpatient parental treatment at the virtual Hospital@Home department, which is part of the Northwest Clinics and provides endocarditis@home treatment.

#### Visceral organ embolism

Five patients experienced visceral embolism, which was detected within 24 h of admission in 2 patients. None experienced overt localising abdominal complaints. One patient also had definite coronary embolism. Three patients had both spleen and kidney embolism. In 4 cases, a streptococcal variant was responsible for the endocarditis of 3 aortic (bio)valves and 2 mitral native valves. One patient had a *Staphylococcus aureus* infection. Two patients also suffered from a spondylodiscitis. No patients were in need of an abdominal or cardiac intervention, and there was no in-hospital mortality in patients with visceral embolism. Three patients were enrolled in outpatient parenteral treatment.

#### Pulmonary embolism

Seven patients had evidence of pulmonary embolisation. Their mean age was 64 ± 9 years, with an equal female/male distribution. None of the pulmonary emboli were observed within 24 h of admission. Of the 7 patients, 6 previously underwent a pacemaker/ICD implantation. Microbiological cultures were positive for *Staphylococcus aureus* (*n* = 2), coagulase-negative* Staphylococcus* (*n* = 1), *Streptococcus variant* (*n* = 1), enterococcus (*n* = 2) and another microorganism (*n* = 1).

Six patients underwent lead and device extraction. None had left-sided distant emboli, while one patient had spondylodiscitis. In-hospital mortality was 1/7, but this was not related to the distant embolisation. Three patients, one with a LifeVest after ICD extraction, were enrolled in successful and uncomplicated outpatient parenteral treatment.

#### Myocardial embolism

In 8 patients, evidence of definite myocardial infarction was found. Six patients experienced chest pain before admission. On average, their maximum troponin I rise was 17,995 ± 17,310 ng/l. Four patients also had discrete ST‑T changes on consecutive electrocardiograms. One patient suffered from ST-elevation myocardial infarction 1 month before hospital admission for endocarditis and had a primary percutaneous coronary intervention of the left descending coronary artery. In retrospect, the aortic valve showed a small, mobile structure suggestive of a vegetation. Four patients previously underwent valve replacement, of whom 2 received a biological aortic valve, 1 bio-Bentall aortic valve and 1 a transfemoral aortic valve.

Four patients underwent cardiac MRI, which confirmed an ischaemic myocardial event. One patient also had radiologic evidence of visceral organ and cerebral embolism. The responsible pathogens were *Streptococci* (*n* = 5) and *Staphylococcus aureus* (*n* = 2); one culture was negative. In-hospital mortality was 3/8: 2 patients died due cardiogenic shock shortly after admission and embolisation and one patient died due to COVID. Four patients were enrolled in successful and uncomplicated outpatient parenteral treatment.

### Endocarditis@home treatment

Organ embolisation did not result in a longer in-hospital stay. The clinical outcome of patients with embolisation was similar to that of patients without embolic events. Of all endocarditis patients, 92 could be treated at home for a mean duration of 25 days; the mean total duration of their hospital stay (including treatment at home) was 47 days (Tab. 2). Two patients died during their treatment at home or at the homecare centre. Both were complex patients without further surgical options who preferred to be treated at home.

**Table 2:** Characteristics of endocarditis patients receiving outpatient parenteral treatment at home

Characteristic	Embolism (*n* = 17)	No embolism (*n* = 75)	*P*-value
Age, years	72 ± 12	74 ± 14	0.829
Female	1 (5)	18 (24)	0.836
**Pathogens**			
*Streptococci*	8	34	0.897
*Staphylococcus aureus*	5	14	0.332
*Staphylococcus epidermidis*	1	4	0.565
*Enterococcus faecalis*	0	7	0.341
Other Gram positives	2	7	0.670
Gram negatives	0	6	0.589
Negative blood cultures	1	4	1.000
**Laboratory findings at hospital arrival**			
C‑reactive protein, mg/l	137	114	0.379
Haemoglobin, mmol/l	8.1	7.0	0.001
Leucocytes, × 10−9/l	13.3	12.3	0.111
Creatinine, μmol/l	146	118	0.025
**Cardiac involvement**			
Aortic valve	8	39	0.713
Mitral valve	5	17	0.543
Tricuspid valve	1	1	0.337
Device infection	4	12	0.485
**Outcomes**			
Total hospital stay duration, days	49	45	0.186
Duration of hospital at home, days	25	25	0.958
**30-day follow-up (*n*** **=** **90)**			
Relapse < 30 days	2	7	0.662
Mortality, total	1	1	0.337
Mortality discharge to 30 days	1	2	0.448

Data are mean ± standard deviation, *n* (%), *n* or mean

## Discussion

This registry-based single-centre experience showed an incidence of distant organ embolisation in daily care of 24%. The auxiliary data registry enabled us to conclude that distant embolisation in patients with infective endocarditis has various ways of presentation: cerebral and coronary embolisation provoke complaints, while visceral emboli remain silent and pulmonary emboli may present with inflammatory signs. Distant organ embolisation was not in itself a contra-indication for outpatient endocarditis@home treatment.

### Incidence of distant organ embolism

This study described the incidence of distant organ embolic events in a non-referred cohort. It has been demonstrated that the true incidence is masqueraded by a lack of comprehensive imaging protocols [10, 11]. The guidelines do not stipulate to which extent embolism should be actively traced [1, 2]. Clinical presentation may alert us of embolisation, but our study results also imply that embolism can occur silently. This is apparent in visceral organ and pulmonary embolisation.

Embolisation to end-arterial vascular beds, such as the cerebral and myocardial vasculature, leads to more recognisable symptoms. The risk of myocardial embolism may be appreciated by determining cardiac troponin levels as first line of exploration. Recent studies have demonstrated that advanced imaging may improve the detection of silent embolic events [1, 2, 12–14]. Further research is needed to develop a multimodality imaging protocol to detect emboli, which occur in 20–37% of endocarditis patients [12–17].

### Relevance of embolism

With regard to the occurrence of distant embolisation, there are three important aspects. Firstly, it may establish the diagnosis of endocarditis. Secondly, it has impact on clinical decision-making in the initial treatment phase. Thirdly, it affects the clinical course of the disease.

Signs of emboli are a minor criterium of the Duke criteria for endocarditis, thereby differentiating between possible and definitive endocarditis. This is valid for intracardiac device endocarditis because lead vegetations may not be visible on echocardiography. PET/CT may also be hampered but will be instrumental to visualise pulmonary micro-emboli [12–14].

Cerebrovascular emboli have a major impact on clinical decision-making. Prominent cerebral malperfusion signs are one of the few reasons to consider acute cardiac surgery, particularly in case of high embolic risk or uncontrolled infections [1, 2, 15–17].

Coronary embolic events are underappreciated [10, 18, 19], but the ESC Guidelines do not mention them [1]. We found a 5% incidence of coronary embolism by angiography once and by cardiac MRI in 4 cases. Our results also underestimated the incidence in a real-life situation due to the absence of clear guidance to ascertain embolisation. In patients with clinically suspicious chest pain, we assessed the troponin I level, albeit a few days after admission. A cardiac MRI study is planned in appropriate patients with troponin I levels > 100 times the upper limit of normal. Systematic review of coronary embolism may be needed in future series to provide appropriate guidance.

Distant embolisation may also be a marker of ongoing local infections or could be the reason that inflammatory parameters such as CRP may again rise and fall during antibiotic treatment [20]. None of our embolic patients had signs of new embolism. A CRP rise is not uncommon after lead extraction resulting from micro-embolism [20].

### Endocarditis@Home treatment

A new treatment modality for endocarditis is outpatient antibiotic treatment [20–23]. The ESC Guidelines provide a schedule for this approach, which would be feasible after the initial 2–3 weeks of the in-hospital phase [1]. We, however, have successfully extended the entry criteria to patients who were recovering from biovalve or device endocarditis as well as *Staphylococcus aureus* infections [20]. Herein, we have described opportunities for stable embolisation patients. One of the prerequisites of this approach is the installation of a solid transmural treatment team that includes nurse practitioners. This way, intravenous antibiotic treatment is safe and may be considered as a third option in the established in-hospital intravenous or even oral treatment of infective endocarditis [24, 25].

### Limitations

We acknowledge several limitations of our study. The retrospective and single-centre design has its shortcomings. However, this study is one of the few studies conducted in a non-surgical hospital, thereby providing information on a non-referred population. Additionally, the relatively frequent use of the PET/CT modality reflected the intense collaboration within the imaging centre and thereby its value for the detection of distant embolisation. Finally, given the diffuse nature and lack of unequivocal definitions, non-organ embolisation was not established.

## Conclusion

This registry-based, single-centre experience showed an incidence of distant organ embolisation in daily care of 24%. Cerebral and coronary embolisation provoked symptoms, while visceral emboli remained silent. Pulmonary emboli may present with inflammatory signs. Distant embolisation was not in itself a contra-indication for outpatient endocarditis@home treatment.
